# Supplementary material for: WordCommentsAnalyzer: A windows software tool for qualitative research
Source: F1000Res. 2018 Sep 4;7:536. Originally published 2018 May 3. [Version 2] doi: 10.12688/f1000research.14819.2 (PMC6137411; doi:10.12688/f1000research.14819.2)
Supplement: Supplementary file 3 [file f1000research-7-17614-s0002.tgz › b2a79496-4ada-47c4-9682-a99ce867836d.zip!/Eight Iranian Health Journals/Yafteh _36.docx]

© Yafteh  . This work is licensed under CC BY 4.0 (https://creativecommons.org/licenses/by/4.0/).

Volume & Issue: دوره 11 - شماره 5

Title: بررسی تاثیر دانه جوانه زده شنبلیله و کلوفیبرات بر تغییرات سطوح سرمی چربیها و ایجاد رگه های چربی ناشی از هیپرکلسترولمی در جدار شریان آئورت خرگوش.

http://yafte.lums.ac.ir/article-1-188-fa.html

: آرترواسکلروز به معنای تجمع چربی و سخت و سفت شدن جدار شریان ها می باشد و تمایل به درگیری نواحی خاصی از سیستم گردش خون دارد که بر اساس ناحیه گرفتار شدت درگیری و ویژگی های مربوط به ضایعه، تظاهرات و شواهد بالینی خاصی را به وجود می آورد. تنها عامل ضروری در پدید آمدن آترواسکلروز، سطح بالای کلسترول LDL است. امروزه از داروهایی نظیر لووستاتین، کلیسترامین و کلوفیبرات جهت کاهش سطح LDL و افزایش HDL استفاده می شود. مواد و روش ها: در این مطالعه اثر مصرف جداگانه و توام دانه جوانه زده شنبلیله و کلوفیبرات بر سطح سرمی چربی های خون و ایجاد رگه های چربی در آئورت 25 خرگوش نر سالم بررسی شده است. خرگوشها به پنج گروه پنج تایی تقسیم شدند و برای مدت 45 روز به ترتیب زیر، رژیم غذایی و دارویی دریافت کردند، گروه یک: رژیم غذایی نرمال بدون دریافت دارو، گروه دو: رژیم غذایی پرچرب بدون دریافت دارو، گروه سه: رژیم غذایی پرچرب به اضافه کلوفیبرات ( 200 میلی گرم دو بار در روز)، گروه چهار: رژیم غذایی پر چرب به اضافه کلوفیبرات (50 میلی گرم دو بار در روز) و گروه پنج: رژیم غذایی پرچرب به اضافه کلوفیبرات (200 میلی گرم دو بار در روز) و پودر دانه جوانه زده شنبلیله (600 میلی گرم دو بار در روز). نمونه گیری از خون حیوانات به صورت ناشتا در ابتدا و انتهای مطالعه انجام شد و از نظر محتوای سرمی چربیها مورد بررسی قرار گرفت. همچنین پس از مطالعه نمونه های پاتولوژی از آئورت حیوانات تهیه و بررسی میکروسکوپی شد. بحث و نتیجه گیری: کلسترول تام، LDL، و تری گلیسیرید در گروههای سه، چهار و پنج در طی مطالعه نسبت به گروه دو افزایش کمتری داشت، در صورتی که افزایش HDL در این گروهها نسبت به گروه دو بیشتر بود. علاوه بر این شدت کمتری از درگیری آئورت با رگه های چربی در گروه پنج دیده شد. نتایج: این نتایج سودمند بودن دانه جوانه زده شنبلیله را هنگام مصرف با کلوفیبرات در پیشگیری و درمان هیپرلیپیدمی و آترواسکلروز در خرگوش مطرح می سازد (P<0.05).

Title: بررسی تاثیر عصاره ریشه گیاه شیرین بیان بر درمان ملاسما

http://yafte.lums.ac.ir/article-1-193-fa.html

: ملاسمانوعی افزایش پیگمانتاسیون پوست شایع و اکتسابی است که به طور عمده در زنان و در مناطق در معرض آفتاب در صورت بروز می نماید. در حال حاضر روش های درمانی مختلفی از جمله داروهای کاهش دهنده پیگمانتاسیون پوست در درمان این بیماری به کار می رود که نتایج درمانی متفاوتی از آنها حاصل شده است. داروی هیدروکینون در حال حاضر به عنوان درمان استاندارد ملاسما شناخته شده است. عصاره شیرین بیان حاوی تعداد زیادی مواد طبیعی مانند Glabridin و Liquiritin می باشد که تاثیرات مفید متعددی روی پوست دارد که از آن جمله می توان از خواص روشن کنندگی پوست نام برد. مطالعه حاضر جهت مقایسه تاثیر درمانی کرم هیدروکینون 4 درصد و کرم عصاره شیرین بیان 4 درصد در درمان ملاسما انجام شده است. مواد و روش ها: این مطالعه بصورت کارآزمایی بالینی روی 38 بیمار زن دچار ملاسما که در فاصله زمانی اردیبهشت 1386 و اردیبهشت 1387 به درمانگاه پوست بیمارستان شهدای عشایر خرم آباد مراجعه و دچار هیپرپیگمانتاسیون دوطرفه و قرینه در ناحیه مالار صورت بودند انجام گرفته است. در این مطالعه کرم هیدروکینون در یک سمت صورت و کرم شیرین بیان در طرف مقابل بصورت یکبار در روز به هنگام شب و به مدت 8 هفته بر روی پوست مالیده شد. مقدار Modified melasma area and severity index (MMASI) برای هر طرف صورت بصورت جداگانه در ابتدای مطالعه و بعد از آن هر 2 هفته تا انتهای مطالعه اندازه گیری شد. آنالیز یافته ها با استفاده از نرم افزار spss و روش های آماری فریدمن Friedman ) )وآزمون رتبه ای علامت ویلکاکسون( Wilcoxon )به عمل آمد. نتایج: 27 تن از بیماران مصرف دارو را تا انتهای مطالعه ادامه دادند.مقدار mmasi در سمتی که باکرم عصاره شیرین بیان درمان گردید از10/6± 59/10 به 17/2± 59/2 و در سمتی که با کرم هیدروکینون تحت درمان بود از34/6± 81/10 به 55/1± 63/1 در پایان 8 هفته کاهش یافت. این نتایج نشان دهنده کاهش معنی دار mmasi در سمت تحت درمان با کرم عصاره شیرین بیان بود (001/0 P< ). با این همه میزان کاهش mmasi در سمت تحت درمان با هیدروکینون به صورت قابل ملاحظه ای بالاتر از آن در مقایسه با سمت تحت درمان باکرم عصاره شیرین بیان بود (002/0 P< ). نتیجه گیری: با اینکه میزان کاهش پیگمانتاسیون ناشی از درمان با کرم عصاره شیرین بیان 4 درصد پس از 8 هفته درمان قابل ملاحظه می باشد (001/0 P< ) ولی کرم هیدروکینون 4 درصد در این مورد به طور قابل توجهی موثرتر از کرم شیرین بیان 4 درصد است (002/0P= ).

Title: بررسی میزان تاثیر اسانس ساتوریا خوزستانیکا بر شدت درد در مبتلایان به پست هرپتیک نورالژیا

http://yafte.lums.ac.ir/article-1-187-fa.html

بیماری زونا در نتیجه فعالیت مجدد ویروس واریسلا زوستر ایجاد می شود. شایعترین عارضه زونا پست هرپتیک نورالژیا (PHN) می باشد.PHN دردی است که بیش از 30 روز از بروز ضایعات پوستی بیماری پایدار بماند.علی رغم وجود درمانهای مختلف، هیچیک درمان قطعی محسوب نمی شوند.برخی از داروهای رایج مانند کاپسیسین منشأ گیاهی دارند. ساتوریا خوزستانیکا که از گیاهان بومی لرستان و خوزستان است دارای اثرات اثبات شده ای در تسکین دردهاست. در این مطالعه تاثیر اسانس ساتوریا خوزستانیکا برPHN بررسی گردید. مواد و روش ها : این مطالعه به صورت کارآزمایی بالینی دوسویه کور روی 50 بیمار مبتلا به PHNانجام شد. بیماران بصورت تصادفی به دو گروه تقسیم شدند. گروه مورد، محلول حاوی اسانس ساتوریا خوزستانیکا با غلظت %5 و گروه شاهد دارونما را دریافت نمودند. شدت درد قبل و بعد از مصرف، در مقیاس عددی ثبت گردید. نتایج با استفاده از آزمون اندازه گیری مکرر، آزمون تی و تی زوجی و من ویتنی تجزیه و تحلیل گردید. یافته ها : میانگین شدت درد در گروه مورد قبل و بعد از مصرف بترتیب 48/1± 12/8 و 9/2±72/5 بود و میانگین شدت درد قبل و بعد از مصرف دارونما در گروه شاهد بترتیب 46/1±04/8 و 52/1±84/7بود که در مقایسه دو گروه، اختلاف معناداری وجود داشت. .همچنین بصورت معناداری پاسخدهی مردان به دارو بیش از زنان بود. %60 بیماران اثرات تسکینی دارو را تجربه نمودند. عوارض جانبی بصورت سوزش و قرمزی در9 بیمار مشاهده شد که همگی در عرض یک ساعت برطرف گردید. اختلاف آماری معناداری داری از نظر جنس و سن بین دو گروه وجود نداشت. بحث و نتیجه گیری : اسانس ساتوریا خوزستانیکا در تسکین PHN موثر است و نسبت به بسیاری از درمانهای موجود از نظر زمان شروع اثر، پایداری اثر، پذیرش از سوی بیماران و عوارض جانبی مناسب تر است.

Title: کاهش نسبی آسیب کلیوی ناشی از سیسپلاتین در موشهای صحرایی از طریق پیش درمانی با عصاره برگ زیتون

http://yafte.lums.ac.ir/article-1-190-fa.html

سیسپلاتین یک داروی مهم ضد نئوپلاسم است که نفروتوکسیسیتی عارضه اصلی آن محسوب میشود و مصرف آن در شیمی درمانی سرطان را محدود می سازد. از آنجائیکه رادیکالهای آزاد اکسیژن تا حد زیادی مسؤول نفروپاتی ناشی از سیسپلاتین هستند، اثرات تجویز خوراکی عصاره اتانولی برگ زیتون (OLE) بعنوان یک آنتی اکسیدان گیاهی بر نفروتوکسیسیتی ناشی از این دارو مورد بررسی قرار گرفت. مواد و روشها: 21 رت نر بالغ به سه گروه تقسیم شدند: گروه OLE75+CP (تجویز خوراکی 14 روزه mg/kg75 OLE قبل از تجویز داخل صفاقی mg/kg5 سیسپلاتین) ، گروهWater +CP (تجویز خوراکی 14 روزه آب قبل از تجویز سیسپلاتین) و گروه Water+Saline (مانند گروه قبلی فقط تجویز سالین بجای سیسپلاتین). نمونه های پلاسما 72 ساعت بعد از تزریق سیسپلاتین جمع آوری شدند و نمونه های ادرار از قبل از خونگیری بمدت 24 ساعت جمع آوری گردیدند. کراتینین و اوره پلاسما، کسر دفع سدیم و پتاسیم، کلییرانس کراتینین و وزن نسبی کلیه ها در گروههای مختلف بعنوان شاخصهای عملکرد کلیه مورد سنجش قرار گرفتند. یافته ها: سیسپلاتین منجر به اختلال معنی دار در همه این شاخصهای عملکرد کلیه شد. تجویز خوراکی OLE باعث کاهش معنی دار کراتینین و کسر دفع پتاسیم شد و کاهش میزان اوره پلاسما در گروه OLE75+CP نسبت به گروه Water+CP در مرز معنی داری بود (08/0p=). سایر شاخصهای عملکرد کلیه بین دو گروه اخیر تفاوت معنی داری نداشتند. بحث و نتیجه گیری: تجویز خوراکی 14 روزه دوز پایین نمونه یی از عصاره برگ زیتون (بطور خاص حاوی اولئوروپئین بالا)، می تواند بطور نسبی نفروتوکسیسیتی ناشی از سیسپلاتین را در رتها کاهش دهد. اثر دوزهای بالاتر عصاره میبایست در تحقیقات بعدی بررسی شود.

Title: مرور سیستماتیک بر مهم‌ترین گیاهان دارویی و جایگاه بین المللی استفاده از آن‌ها در درمان بیماری‌ها

http://yafte.lums.ac.ir/article-1-186-fa.html

: شناخت خواص درمانی گیاهان دارویی و همچنین مضرات درمان‌های نوین منجر به تشویق مردم به استفاده از گیاهان دارویی شده است. مطالعات بسیاری در زمینه گیاهان دارویی خاص و کارایی آن‌ها در درمان بیماری‌های مشخص انجام و مقالات زیادی در این رابطه منتشر شده است. اما در این بین وجود مطالعه‌ای که مروری بر مطالعات مذکور باشد احساس می‌شود که هدف این مطالعه می‌باشد. مواد و روش‌ها: با استفاده از کلمات کلیدی herbal medicine، herb medicine، phytotherapy و extract medicine تعداد 7862 مقاله چاپ شده از سال 2000، در پایگاه‌های اطلاعاتی الکترونیکی PubMed، ScienceDirect و Springer جستجو و مطالعه شد. بیماری، گیاه مورد استفاده و نظر نویسنده در مورد کارایی گیاه به کار گرفته شده، مشخص گردید. در پایان این نتایج مورد تجزیه و تحلیل آماری قرار گرفتند. یافته‌ها: 5/11% کل مقالات در ارتباط با 8 گیاه بودند که به ترتیب فراوانی عبارتند از Ginseng (عشقه)، Salvia L. (مریم گلی)، Ginkgo biloba (ژینکو)، ypericum L. (گل راعی)، Allium L. (سیر)، Zingiber (زنجبیل)، Aloe vera (صبرزرد) و Marijuana(شاهدانه). شایع‌ترین بیماری‌های مورد بررسی شامل بیماری‌های عصبی– روانی، ایمنی- التهابی، گوارش، قلبی – عروقی و سرطان بودند. در2/89% از مقالات به اثرات مثبت و در اندکی از آن‌ها به عدم مزایای استفاده از گیاهان در درمان اشاره شده است. 6/54% مقالات بر لزوم انجام تحقیقات بیش‌تر برای استفاده درمانی از گیاهان تاکید داشتند. بحث و نتیجه‌گیری: با توجه به این گیاهان دارویی متداول‌ترین روش طب مکمل در سراسر جهان است و اکثر مطالعات انجام شده به اثرات مثبت آن اشاره دارند، توجه بیشتر برای انجام مطالعات علمی دقیق‌تر برای تعیین مکانیزم اثر آن‌ها ضروری است.

Title: بررسی عرضه و مصرف گیاهان دارویی موجود در عطاری‌های استان لرستان در سال 1387

http://yafte.lums.ac.ir/article-1-191-fa.html

از آنجا که برآیند فعالیت های مرتبط با صنعت گیاهان دارویی در راستای حفظ و ارتقاء سلامت جامعه قرار می گیرد، توجه به اهمیت وضعیت گیاهان دارویی موجود در عطاری ها، تعیین گونه های مختلف، میزان مصرف و مناطق عرضه کننده به عنوان اصلی ترین عوامل در بازار گیاهان دارویی، می تواند راهنمای خوبی برای برنامه ریزان و سیاست گذاران صنعت دارویی کشور و استان لرستان که یکی از مهمترین رویشگاه های این گیاهان در کشور است، باشد تا با برنامه ریزی مناسب علمی شاهد اصلاح فرهنگ مصرف گیاهان دارویی و از همه مهمتر ارتقاء سلامت در استان لرستان باشیم. مواد و روش ها: نوع مطالعه در این پژوهش مقطعی است و تکنیک مورد استفاده پرسشنامه سه بخشی است که ویژگی های اقتصادی- اجتماعی، تعداد و انواع گیاهان دارویی و خواص دارویی آنها را مورد سنجش قرار می دهد. جامعه آماری این پژوهش را، کلیه عطاری های استان لرستان تشکیل می دهند. روش نمونه گیری از نوع نمونه در دسترس با حجم نمونه 69 باب می باشد. یافته ها: نتایج نشان داد که تعداد کل گیاهان دارویی موجود در عطاری ها حدود 336 گونه و میانگین کل وزن فروش 48/128 کیلوگرم در سال است. اکثر گیاهان دارویی(5/94 درصد) از خارج از استان وارد می شوند و تنها 5/5 درصد آنها در استان لرستان تهیه و در اختیار عطاری ها قرار می گیرند. نتیجه گیری: وضعیت خاص آب و هوای استان لرستان و اقلیم متنوع آن سبب تنوع چشمگیر پوشش گیاهی استان شده است. با این حال نتایج حاصل از این پژوهش نشان می دهد که مناطق عمده تأمین کننده گیاهان دارویی خارج از استان می باشند. به نظر می رسد که می توان با برنامه ریزی مناسب در زمینه کشت گیاهان دارویی در استان شاهد اشتغالزایی بیشتر و عرضه فرآورده های این محصولات به عطاری های استان لرستان و سایر استان ها باشیم. و حتی به صادرات فرآورده های گیاهی تولید شده در استان به سایر کشورها بیندیشیم.

Title: بررسی ترکیبات فرار در گلبرگ و پیکره رویشی و استحصال روغن بذر گاوزبان اروپایی توسط روش Cold Press

http://yafte.lums.ac.ir/article-1-185-fa.html

: با توجه به بررسی های صورت گرفته تحقیقات اندکی در مورد ترکیبات فرار گیاه گاوزبان اروپایی گزارش شده است. هدف از انجام این تحقیق بررسی ترکیبات فرار موجود در پیکره رویشی، گلبرگ و روغن بذر این گونه در استان لرستان می باشد. مواد و روشها: استخراج اسانس از گلبرگ ها به روش تقطیر با آب مقطر و با دستگاه کلونجر انجام شد. آنالیز و شناسایی ترکیبات فرار گلبرگ با استفاده از دستگاه GC/MS صورت پذیرفت. استخراج و شناسایی ترکیبات فرار پیکره رویشی با استفاده از روش جدید SPME صورت گرفت. استخراج روغن بذر با استفاده از روش Cold-Press انجام شد. سپس شناسایی و اندازه گیری ترکیبات روغن توسط دستگاه GC/MS انجام گردید. یافته ها: در گلبرگ ها فقط ترکیب کارواکرول، در پیکر رویشی سه ترکیب بتا یونن، تترادکان و دکانال و در روغن بذر گاوزبان 16 ترکیب استخراج و شناسایی شد. مهمترین ترکیبات شیمیایی تشکیل دهنده روغن بذر گاوزبان اروپایی شامل، ان ان- دی متیل اتانول آمین، بتا دی گلیکوزید3-6-گلوکورونو متیل، بنزآلدهید- 4 متیل، 3-هیدروکسی تترافوران، هگزا دکانوئیک اسید، هپتانوئیک اسید، گاما بوتیرولاکتون و اتیل اکتا دک 9- انوات می باشند. این ترکیبات 63.4 درصد از کل ترکیبات روغن بذر را تشکیل داده است و 7 ترکیب باقی مانده فقط 9.5 درصد از کل ترکیبات شیمیایی روغن بذر را شامل می شود. همجنین یک ترکیب ناشناخته (27.1 درصد) در روغن بذر گاوزبان اروپایی شناسایی گردید. بحث و نتیجه گیری: با توجه به نتایج به دست آمده از این تحقیق مواد فرار درصد کمی از ترکیبات گلبرگ و پیکره رویشی گیاه دارویی گاوزبان اروپایی را تشکیل داده است. همچنین روش Cold-Press یک روش سریع و ساده برای استخراج و شناسایی ترکیبات شیمیایی روغن بذر گیاهان می باشد.

Title: شناسایی برخی گونه‌های قارچ های ماکروسکوپی دارویی و سمی شهرستان خرم‌آباد

http://yafte.lums.ac.ir/article-1-192-fa.html

جمعیت و تنوع قارچ‌ها در شرایط اکولوژیک مناطق مختلف بسیار متغیر است. این موجودات منبع مواد فوق‌العاده با ارزشی مانند آنتی‌بیوتیک‌ها بوده و از دیر باز قارچ‌های کلاهک‌دار به عنوان دارو در طب سنتی استفاده شده اند. با توجه به مصارف مختلف قارچ‌ها در صنایع داروسازی، شناسایی آنها اولین گام در بهره‌برداری دارویی از این موجودات است. وجود گونه‌های سمی در بین قارچ‌های وحشی سبب ایجاد مسمومیت‌های خطرناک وگاهی مرگ آور می‌شود. هدف از این بررسی شناخت قارچ ها و معرفی آنها به مردم و در نتیجه کاهش مشکلات حاصل از مسمومیت ناشی از خوردن قارچ‌های سمی و کمک به پزشکان درتشخیص مسمومیت و تجویز درمان مناسب است. هدف از شناسایی قارچ‌های دارویی نیز استفاده از پتانسیل موجود در داروسازی است. مواد و روش‌ها: در بهار و پاییز سال 1387 و 1388 نمونه های از قارچ های کلاهک‌دار در شهرستان خرم آباد جمع آوری و مشخصات مورفولوژیک ماکروسکوپی و میکروسکوپی آنها در آزمایشگاه بررسی و با منابع علمی موجود مطابقت داده شد. یافته ها: 8 گونه قارچ دارویی و سمی شناسایی گردید که شامل گونه maculate Collybia ؛ گونه Coprinus atramentarius؛ سه گونه از جنس Boletus شامل (B luridus، felleus .B و (B. satana؛ دو گونه از جنس Lactarius شامل (L. vellereus و L. piperatus ) و گونه Hypholoma capnoides است. بحث و نتیجه گیری: با توجه به وجود چنین تنوعی در این منطقه، می توان از اطلاعات بدست آمده بهره برداری های زیادی در کاربردهای پزشکی نمود.

Title: گیاهان دارویی استان لرستان

http://yafte.lums.ac.ir/article-1-184-fa.html

این پژوهش در واحد گیاهان دارویی و محصولات فرعی، مرکز تحقیقات منابع طبیعی و امور دام استان لرستان به مدت 6 سال به اجرا درآمد. هدف از این کار جمع‌آوری و شناسایی گیاهان دارویی موجود در استان لرستان، تعیین رویشگاهها، پراکنش،فرم رویشی، استفاده‌های سنتی، اندام مورد استفاده، روش استفاده، ویژگی‌های گیاه شناسی،نام محلی، نام فارسی و نام علمی گیاهان مذکور بوده است. مواد و روشها: در همین راستا اقدام به استخراج لیست گیاهان دارویی گزارش شده از استان در منابع مختلف، شناسای افراد بومی صاحب نظر گیاه درمانی در استان و در نهایت جمع آوری و شناسایی گیاهان از سطح استان گردید. یافته‌ها: در مجموعً 151 گیاه دارویی از سطح استان جمع آوری و شناسایی گردید، که این گیاهان متعلق به 63 تیره و 90 جنس می‌باشند، بیشترین گونه‌های جمع آوری شده متعلق به تیره هایLabiatae, Compositae, Legominosae , Liliaceae Rosaceae , و Umbelliferae است. بحث و نتیجه‌گیری: طبق بررسیهای بعمل آمده در منابع مختلف 96 گونه گیاهان دارویی از استان گزارش گردیده بود. که در طی این تحقیق 151 گونه گیاه دارویی از سطح استان جمع‌آوری و شناسایی گردید. در مقایسه با گیاهان دارویی جمع‌آوری شده در استان‌های بوشهر 70 گونه (7)، هرمزگان 113 گونه (8)، مرکزی 144 گونه (9)، کردستان 144 گونه (10)، تنوع بیشتری نشان می‌دهد اما در مقایسه یا استان‌های زنجان 163 گونه(11)، همدان 315 گونه (12)، قزوین 250 گونه (13)، به نظر می‌رسد که از تنوع کمتری برخوردار است.

Title: تعیین ارزش غذایی و کیفی ارقام مختلف انجیر استان لرستان

http://yafte.lums.ac.ir/article-1-198-fa.html

انجیر از جمله اولین گیاهانی است که بوسیله انسان ها مورد کشت و کار قرار گرفت و بطور گسترده به صورت خشک و تازه مورد مصرف قرار می گیرد. با توجه به عدم وجود اطلاعات در مورد ارزش غذایی ارقام مختلف انجیر استان لرستان این تحقیق در دانشگاه علوم پزشکی استان لرستان در سال 1388 انجام گرفت. مواد و روش ها: در این تحقیق مقدار عناصر (پتاسیم، کلسیم، فسفر، آهن و روی)، پروتئین، چربی، قند، بریکس (مواد جامد محلول)، اسیدیته، pH، ویتامین ث، نسبت بریکس به اسیدیته و سفتی در سه رقم انجیر متداول در استان لرستان (سیاه، رشه و سفید) و در مناطق خرم آباد (گوشه)، کلات زیودار، معمولان و زوران تل زیودار مورد سنجش قرار گرفت. یافته ها: نتایج حاصل از این تحقیق نشان می دهد که مناطق مختلف انجیرکاری در استان لرستان (گوشه، کلات زیودار، معمولان و زوران تل زیودار) پتانسیل تولید میوه های با کیفیت های متفاوتی را دارند که در بین رقم های کاشته شده در این مناطق رقم سیاه بعنوان یک رقم با کیفیت معرفی می گردد. بحث و نتیجه گیری: مقایسه بین غلظت عناصر در انجیر در این تحقیق با سیب بعنوان یک میوه متداول نشان می دهد که مقدار عناصری مثل کلسیم ، آهن، فسفر، پتاسیم و روی به مراتب بالاتر از غلظت آنها در میوه سیب می باشد. اما تنوع داده ها در سایر مقالات در مورد انجیرو همچنین تحقق حاضر حاکی از آن است که این گیاه با رسیدگی مناسب توانایی دارا بودن مقادیر بیشتری از این عناصر و فاکتورها را داراست.

Title: تاثیر اسیدهای چرب ضروری روغن ذرت و بادام زمینی بر روند اسپرماتوژنز در موش صحرایی

http://yafte.lums.ac.ir/article-1-197-fa.html

دانه های روغنی با داشتن اسیدهای چرب غیر اشباع و اشباع بر روی فرآیندهای اسپرماتوژنز در بیضه موثرند. مواد و روشها: در این تحقیق از 21 عدد موش صحرایی نر تازه از شیر گرفته شده، هر گروه ( کنترل، روغن ذرت، روغن بادام زمینی) به تعداد 7 موش در یک قفس به مدت سه ماه استفاده شد. تغذیه موش ها بصورت 10 درصد (w/w) غذای روزانه از روغن ذرت و روغن بادام زمینی در گروهای درمان صورت گرفت. پس از رسیدن به مرحله بلوغ با مشاهدات ماکروسکوپی در پایان 3 ماهگی، آزمایشات هورمون شناسی، مقاطع بافتی از بیضه تهیه شده و بررسی اندازه گیری های وزنی و حجم بیضه در هر 3 گروه از موش ها صورت گرفت. یافته ها: نتایج نشان داد که کاهش معنی داری در میزان هورمون تستسترون و FSH درگروههای درمان نسبت به کنترل دیده میشود((P<0.001 در حالیکه این تفاوت معنی دار در مورد هورمون LH و حجم بیضه در گروه روغن ذرت ( (P<0.001 و در گروه روغن بادام زمینی (p< 0.05) نسبت به گروه کنترل می باشد، هم چنین کاهش وزن بیضه در گروههای درمان نسبت به کنترل مشاهده گردید اما این تفاوت معنی دار نبود. بحث و نتیجه گیری: در مجموع مطالعه حاضر نشان میدهد که مصرف روغن ذرت و بادام رمینی احتمالا بخاطر وجود اسیدهای چرب غیر اشباع بویژه آراشیدونیک اسید که به نوبه خود پیش ساز پروستا گلندین ها می باشد می توانند با تا ثیر بر روی متابولیسم پایه و افزایش فعالیت انسولین در بافت بیضه باعث ازدیاد تکثیر سلولی در لایه اسپرماتوگونی لوله های سمینفر و افزایش روند اسپرماتوژنز شده اما به دنبال آن موجب ایجاد توقف در تبدیل اسپرماتوسیت به اسپرماتوزوئید( مرحله میوتیک اسپرماتوژنز) در لوله های سمینفر گردند.
